# Pathogenesis and biomarkers of natural killer T cell lymphoma (NKTL)

**DOI:** 10.1186/s13045-019-0717-6

**Published:** 2019-03-15

**Authors:** Nagavalli Somasundaram, Jing Quan Lim, Choon Kiat Ong, Soon Thye Lim

**Affiliations:** 1 Division of Medical Oncology, National Cancer Centre Singapore, 11 Hospital Drive, Singapore, 169610 Singapore; 2 Singhealth Duke- NUS Blood Cancer Centre, Singapore, Singapore; 3 Duke-NUS Medical School, Singapore, Singapore; 4 Lymphoma Genomic Translational Research Laboratory, Division of Cellular and Molecular Research, National Cancer Centre Singapore, 11 Hospital Drive, Singapore, 169610 Singapore; 5 Genome Institute of Singapore A*STAR, Singapore, Singapore

**Keywords:** NK/T cell lymphoma, Pathogenesis, EBV, JAK/STAT, PD1, PDL1

## Abstract

Natural killer T cell lymphoma (NKTL) is an aggressive disease with very poor treatment outcomes in the advanced stages. With chemotherapy, initial response rates to treatment are high but responses are short lived. A better understanding of the complex molecular pathogenesis of this disease is essential in order to design and develop better therapeutics with improved efficacy. This review aims to summarise the key pathogenic mechanisms in NKTL which may have significant prognostic and therapeutic implications.

NK T cell lymphoma (NKTL), or extranodal NK T cell lymphoma, nasal type as classified by the World Health Organisation (WHO), is a non-Hodgkin lymphoma that has a predilection for the upper aerodigestive tract and can involve other non-nasal sites such as the gastrointestinal tract, skin, soft tissue and testis [1]. This is an aggressive disease with an Asian and Latin American preponderance [2–4]. This disease is characterised immunophenotypically by positivity for CD2, CD56, cytoplasmic CD3ε and cytotoxic molecules such as granzyme B and  TIA1. Demonstration of EBV-encoded RNA (EBER) is a prerequisite for the histological diagnosis of NKTLs. Early stages of the disease are treated with radiotherapy or a combination of chemoradiation with good clinical outcomes. However, in advanced stages, this disease is invariably fatal despite the initial good responses with multi-agent chemotherapy regimens.

Natural killer (NK) cells derive from the lymphoid lineage, together with B and T cells. While NK cells have traditionally been classified as a component of the innate immune system, they have been shown to have characteristics of adaptive immunity such as antigen specificity, immunologic memory and ability to undergo clonal expansion when exposed to a pathogen. NK cells, similar to its T cell counterpart, arise from a common lymphocyte precursor in the bone marrow. Mature NK cells can be found in multiple organs such as the spleen, liver, lung and blood [5, 6].

In recent years, the availability of genome sequencing technologies has changed the diagnostic and therapeutic paradigm in many diseases. NKTL is no exception—the understanding of molecular pathogenesis of NKTL has moved leaps and bounds [7]. Figure 1 provides an overview of our current understanding of the key biologic mechanisms that drive this disease. A discussion of the various pathogenic mechanisms will be presented in this review.

**Fig. 1:**
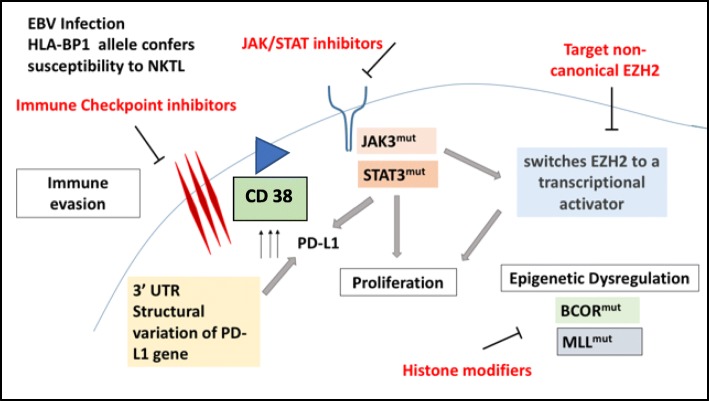
Summary of the pathways involved in the pathogenesis of NKTL. This figure summarises the key pathways that are involved in the pathogenesis of NKTL, namely, mechanisms of immune evasion, JAK/STAT pathway alterations, CD38 expression and epigenetic dysregulation

## EBV infection

Epstein-Barr virus (EBV) infection plays a crucial role in the pathogenesis of NKTL, though the actual mechanism remains to be understood. The identification of EBV genomes in sequencing data or the immunohistochemical stains of EBV non-coding RNA (EBER) in NKTL samples provided initial hints of the oncogenic role of EBV in this disease. A causative relationship between EBV and NKTL was established based on the presence of clonal and episomal forms of EBV in tumour cells, in addition to EBV-encoded proteins [8, 9]. In NKTL, EBV-host integration sites were found in various repeat families of the human genome, such as SINE, LINE and satellite [10]. Notably, a 109 bp long EBV genomic fragment was found to integrate into the intron of the human non-homologous end-joining factor 1 (NHEJ1) gene, which led to the downregulation of the gene transcripts [10]. NHEJ1 is a DNA repair factor essential for the NHEJ pathway, which preferentially mediates repair of double stranded breaks (DSBs). Failure to repair DSBs would result in genome-wide instability that could lead to the onset of NKTL.

Infection by EBV induces expression of genes encoding nuclear antigens, membrane proteins and non-coding RNAs in B cells, all of which induce cell proliferation and transformation into lymphoblastoid cell lines. However, in order to escape from T cell mediated immune responses, the infected B cells enter into a latent state. The pattern of gene expression in the various latent phases has been associated with the different EBV-related malignancies. Latency phase II, with expression of EBNA1, LMP1, LMP2A and LMP2B, has been the most implicated in NKTL, in addition to nasopharyngeal carcinoma and Hodgkin lymphoma. The expression of these genes plays an important role in these tumours by modulating cell signalling and forming barriers to apoptotic signals. However, among the Latency II tumours, different mechanisms drive the various gene expressions, contributing to the unique characteristics of each of these malignancies [11]. A novel transcript LMP2 TR was identified at high levels in NKTL, resulting in a high level of expression of LMP2B in the absence of LMP2A. With this, LMP2B has been postulated to play an important role in the pathogenesis of NKTL and may serve as a potential therapeutic target [12]. Consistently, we observed that most of the latent genes were expressed in NKTL, including those of EBNA family, LMP family and BARTs. The BART RNAs are a heterogeneously spliced group of EBV RNAs transcribed rightward from position 138352 to 160531 on the EBV wild-type genetic map [13–15]. Comparatively, this region is highly transcribed and it consists of about 20 microRNA and the putative proteins RPMS1 and A73 [10]. This highly transcribed region of the EBV genome most probably has an important regulatory role on itself and the host genome, in driving the disease and immune evasion, which warrant further investigation.

## Genetic associations in NKTL

The genetic factors that predispose individuals to NKTL were not known until recently. Our genome-wide association study (GWAS) in NKTL identified a common SNP (rs9277378) in the HLA-DPB1 allele which conferred a 2.3 times higher risk of NKTL compared to baseline [16]. The four amino acids within HLA-DPB1, namely Gly84-Gly 85-Pro86-Met87, play a crucial role in CD4 T lymphocytes for antigen presentation, hence contributing to its hereditary susceptibility to NKTL. This study also demonstrated that the HLA locus associated with NKTL is distinct from other EBV-driven malignancies such as nasopharyngeal carcinoma and Hodgkin lymphoma. Besides distinct differences in host genetic susceptibility, we have recently demonstrated that different EBV strains predominate among these diseases [10]. These findings imply that these diseases are driven by distinct biological mechanisms. However, the potential implications of different EBV strains with the host genome for these diseases are still unclear and require further investigation.

Familial NKTL is a rare phenomenon. At the time of this writing, only two sets of familial NKTL cases have been reported. The earlier report was on a father-son pair with known heavy exposure to pesticides, and no specific genetic element was evident in their tumours [17]. In the later report, a novel recessive and homozygous germline mutation in *FAM160A1* was found in the tumours of two male siblings from a non-consanguineous Chinese family [18]. In this recent case report, *FAM160A1* was also found to be overexpressed in these patients’ tumours harbouring the mutant *FAM160A1* when compared to sporadic NKTL tumours. Interestingly, *FAM160A1* was found to be expressed predominantly in the CD68-positive histiocytes rather than the lymphomatous cells, which suggests that the mutant *FAM160A1* might play a role in the pathogenesis of this disease by altering the microenviromnent of the tumour.

## JAK/STAT pathways

The Janus kinase/signal transducers and activators of transcription (JAK/STAT) pathway is crucial to haematopoiesis and immune development, in addition to other essential functions. Using targeted sequencing of 188 genes associated with the JAK/STAT pathway approach, we have recently demonstrated that alteration of this pathway is highly prevalent (73%) in peripheral T-cell lymphoma (PTCL) and NKTL [19]. In this study series, *STAT3* was identified to be the most frequently mutated gene followed by *TP53*, *JAK3*, *JAK1* and *SOCS1* of the JAK/STAT cascade, suggesting that targeting this pathway might benefit a large portion of NKTL patients. The prevalence of *STAT3* mutations in NKTL was reported to range from 8 to 27% in various studies [19–23].

In addition to the frequent STAT3 activating mutations, *JAK3* activating mutations were also identified in NKTL, in 34% of cases [24]. An additional study reported novel *JAK3* mutations, residing in the pseudokinase domains of *JAK3* [25]. The frequency of *JAK3* mutations varies across different populations, ranging from 0 to 35% [26, 27]. Functional overexpression of *JAK3*^*A572V*^ resulted in the phosphorylation of downstream effector proteins, STAT3 and STAT5. Importantly, preclinical study with Tofacitinib, a pan-JAK inhibitor, could effectively reduce tumour growth and metastatic spread of NKTL [26] indicating that JAK3 is a promising therapeutic target for NKTL. A novel JAK3-specific inhibitor (PRN371) was further developed, to supersede tofacitinib in terms of specificity and durability in inhibiting JAK3 in NKTL [28]. However, the single-agent regime with PRN371 did not confer complete response in mice. While this observation could partially be attributed to suboptimal dosing or delivery of the drug to the target tumour site, this could also reflect the complexity of the JAK/STAT signalling pathway that has yet to be understood fully and hence may warrant combination therapeutic approaches for improved efficacy.

Activating *STAT3* mutations was also demonstrated to confer resistance to PRN371 by rescuing the inhibition STAT3/5 phosphorylation in NKTL [28] suggesting that the activation of *STAT3* could be a biomarker of resistance to JAK3-inhibition therapy. In activated B cell subtype of diffuse large B cell lymphomas (DLBCL-ABC), in vivo inhibition of STAT3 was found to be a more effective strategy in suppressing tumour growth than targeting upstream JAK inhibition [29]. Hong et al. demonstrated that AZD9150, an antisense oligonucleotide (ASO) inhibitor of STAT3, achieved an almost complete inhibition of STAT3 in lymphoma and lung patient-derived explant models [30]. This provided the proof-of-concept that STAT3, a notoriously difficult protein to inhibit therapeutically, can now be better positioned for better treatment outcome.

Another interesting role of STAT3 in NKTL is its relationship with immune surveillance and evasion. We demonstrated that activated STAT3 could upregulate *PD-L1* by binding directing to the proximal promoter of *PD-L1* [19] Inversely, blocking STAT3 with ASO or Statiic downregulated PD-L1 expression effectively. It was further shown that NKTL tumours with high expression of phosphorylated STAT3 correlated significantly with PD-L1 levels highlighting its clinical importance in immune checkpoint inhibition. STAT3 contributes to tumour immune evasion through the accumulation and activation of tolerogenic dendritic and Treg cells, as well as the upregulation of immune checkpoint proteins such as CTLA-4, programmed cell death protein 1 (PD-1), and programmed death ligand 1 (PD-L1) [31]. PD-L1 expression in NK/T cell lymphoma has been reported to be 56–93% in various studies and this has stirred excitement in the use of PD-1/PD-L1 inhibitors in NKTL [19, 32–34]. These studies suggest that inhibiting STAT3 could effectively challenge the survivability of NKTL by simultaneously disrupting its immune evasion pathway. A combination strategy, using PD1/PDL1 antibodies and STAT3 inhibitors, may be yet another therapeutic strategy that can be explored.

## *PD-L1* structural variations

Anti-PD1 therapy has shown promising activity in relapsed/refractory (R/R) NKTL [35]. This was echoed in another study that demonstrated 57% response rates in R/R NKTL with pembrolizumab (PD1 blockade) [36]. In an attempt to understand the mechanism of response or resistance, we performed whole genome sequencing on the tumour of 11 NKTL patients treated with pembrolizumab [37]. Our data revealed that *PD-L1* structural variations (SV) in the 3′UTR region of the *PD-L1* gene were seen exclusively in 4 out of 7 patients who achieved complete response (CR) to pembrolizumab (PD-1 antibody) and not seen in any of the non-responders. Structural variation in 3′UTR region of the PD-L1 gene was also demonstrated in a patient with chemorefractory ovarian cancer who had CR to pembrolizumab [38]. While these findings need to be validated in a larger dataset, this illustrates a molecular mechanism that would explain the efficacious activity of anti-PD-1 therapy in NKTL patients.

In the same study, we have also identified recurrent *JAK3*-activating mutations in two of the seven patients who had achieved CR to pembrolizumab [37]. *JAK3* mutations were also seen in one patient who had prolonged benefit from anti-PD-L1 therapy in refractory lung adenocarcinoma [39]. JAK3 activation deregulated the cytokine receptor signal transduction in lung cell lines, led to the upregulation of PD-L1, and provided the explanation to the durable response seen in this patient with anti-PD-L1 blockade therapy. Taken together, *JAK3*-activating mutation and PD-L1 3′UTR SV are potential biomarkers which could better select NKTL patient for immune checkpoint blockade therapy.

## Epigenetic dysregulation

### EZH2

Enhancer of zeste homologue 2 (EZH2) is a H3K27-specific histone methyltransferase and a catalytic subunit of the polycomb repressive complex 2 (PRC2) molecule. EZH2 is aberrantly expressed in NKTL, at both the protein and mRNA levels, as opposed to normal NK cells. [40, 41]. MYC activation has been shown to play a significant role in EZH2 overexpression by suppression of its negative regulatory microRNAs [41, 42].and its canonical oncogenic function is to transcriptionally silence the expression of tumour suppressor genes with its histone methyltransferase. One such example is the suppression of tumour suppressor gene TNFAIP3/A20 mediated by EZH2 histone methyltransferase, thereby activating NFkB pathway and resulting in resistance to apoptosis in NKTL cell lines [43]. In NKTLs, EZH2 has also been shown to an additional PRC2 complex non-canonical function—a transcriptional coactivator attributed from genome-wide decreased H3K27me3 levels. The phosphorylation of EZH2 by JAK3 results in the dissociation of the PRC2 complex, lifting H3K27me3 epigenetic markers off the genome, hence giving EZH2 its non-canonical role of being a transcriptional coactivator in NKTL [44]. Targeting EZH2 has gained interest in recent years and some of the therapeutics have been summarised in Table 1.

**Table 1:** This table describes the genes implicated in the pathogenesis of NKTL and the potential therapeutic targets

Gene	Therapeutic target	Comments
JAK3	Tofacitinib	First in class JAK 3 inhibitor. Single-agent JAK3 inhibition did not result in CR in NKTL cell lines [28]. Combination strategies may need to be explored
STAT3	AZD9150—antisense oligonucleotide inhibitor [30]	AZD9150 demonstrated promising activity in preclinical and clinical studies in lymphoma. Given that STAT3 activation leads to high PDL1 expression, combination of STAT3 and PD1/PDL1 inhibitors may be explored [19]
EZH2	DZNEP [43]	Non-specific EZH2 inhibitor that had significant toxicities in animal models
	Tazemetostat [50]	Phase 2 study shows promising activity in follicular lymphoma, especially in EZH2 mutated patients
	SHR2554	Selective small molecule inhibitor of the EZH2 histone-lysine methyltransferase ongoing phase 1 study in refractory lymphomas (NCT03603951)
	EPZ005687 [51]	Reduces H3K27 methylation in various lymphoma cell lines
	GSK126 [52]	Competitive small molecule inhibitor of S-adenosylmethionine. Has been tested in Diffuse large B cell lymphoma (DLBCL) and myeloma cell lines [52] and DLBCL xenograft models [53]
PD 1 inhibition	Pembrolizumab [35, 36]	PD 1 inhibitors have demonstrated promising activity in relapsed/refractory NKTL. Potential biomarkers that may allow better selection of patients for PD-1 inhibitors need to be explored
	Nivolumab [54]	
CD38	Daratumumab [55]	Interim analysis of phase 2 study demonstrated 35.7% response in NKTL. CD38 status did not correlate with responses

### BCOR and MLL

BCOR, also known as BCL6 co-repressor, is another epigenetic modifier and it forms a component of the PRC1 complex [45]. The prevalence of BCOR mutations in various studies has been shown to be from 12 to 32% in NKTL [22, 23]. These mutations result in loss of BCOR function. EBV-positive tumours, including solid tumours, have been noted to harbour BCOR mutations suggesting that BCOR mutations may be an important aspect of EBV-related pathogenesis.

MLL2 is an epigenetic regulator which plays a crucial role in cell development and metabolism [46]. Its function as a tumour suppressor has been described as well [47]. Varying frequency of MLL2 mutations has been reported, ranging from 6.7 to 80%. However, the functional implications of this mutation in NKTL remain to be understood.

## Other contributing factors to NKTL pathogenesis

### DDX3X

Alterations in DDX3X gene are another mechanism contributing to the pathogenesis of NKTL. DDX3X is a gene located on the X chromosome, and alterations in this gene result in disruption of its RNA-unwinding function and suppression of cell proliferation through interactions between NFkB and MAPK pathways. DDX3X alterations were found at a frequency of 12% and 20% of NKTL cases from Japan and China, respectively, and have been associated with advanced stage disease and poor outcomes [21].

### CD38

CD38 is a transmembrane glycoprotein which is strongly expressed in NKTL. Wang et al. demonstrated that CD38 is expressed in majority of NKTL with strong expression being seen in almost 50% of the NKTL population. Strong CD38 expression was associated with poor treatment outcomes [48]. Daratumumab is a novel anti CD38 human monoclonal antibody that has shown to have promising activity in preclinical models. Complete remission was reported in one heavily pretreated patient with advanced NKTL, resulting in further ongoing trials to assess efficacy of this strategy in this disease [49].

## Conclusions

The recent data on the various driving mechanisms behind NKTL attempt to unravel the complex pathogenesis driving this disease, which at present has poor treatment outcomes. A summary of current therapeutic targets is presented in Table 1. With a better understanding of the underlying pathogenic mechanisms, biologically sound therapeutic strategies can be employed to potentially overcome this disease.

## Abbreviations

EBER: EBV non-coding RNA
EBV: Epstein-Barr virus
EZH2: Enhancer of zeste homologue 2
JAK/STAT: Janus kinase/signal transducers and activators of transcription
NK: Natural killer
NKTL: Natural killer T cell lymphomas
PD-1: Programmed cell death protein 1
PD-L1: Programmed death ligand 1
WHO: World Health Organisation

## References

[1] Swerdlow SH, Campo E, Pileri SA, Harris NL, Stein H, Siebert R (2016). The 2016 revision of the World Health Organization classification of lymphoid neoplasms. Blood.

[2] Aozasa K, Takakuwa T, Hongyo T, Yang WI (2008). Nasal NK/T-cell lymphoma: epidemiology and pathogenesis. Int J Hematol.

[3] Aozasa K, Zaki MA (2011). Epidemiology and pathogenesis of nasal NK/T-cell lymphoma: a mini-review. ScientificWorldJournal.

[4] Haverkos BM, Pan Z, Gru AA, Freud AG, Rabinovitch R, Xu-Welliver M (2016). Extranodal NK/T cell lymphoma, nasal type (ENKTL-NT): an update on epidemiology, clinical presentation, and natural history in north American and European cases. Curr Hematol Malig Rep.

[5] Sun JC, Lanier LL (2011). NK cell development, homeostasis and function: parallels with CD8(+) T cells. Nat Rev Immunol.

[6] Geiger TL, Sun JC (2016). Development and maturation of natural killer cells. Curr Opin Immunol.

[7] de Mel S, Soon GS, Mok Y, Chung TH, Jeyasekharan AD, Chng WJ (2018). The Genomics and molecular biology of natural killer/T-Cell lymphoma: opportunities for translation. Int J Mol Sci.

[8] Cai Q, Chen K, Young KH (2015). Epstein-Barr virus-positive T/NK-cell lymphoproliferative disorders. Exp Mol Med.

[9] Fox CP, Shannon-Lowe C, Rowe M (2011). Deciphering the role of Epstein-Barr virus in the pathogenesis of T and NK cell lymphoproliferations. Herpesviridae.

[10] Peng RJ, Han BW, Cai QQ, Zuo XY, Xia T, Chen JR, et al. Genomic and transcriptomic landscapes of Epstein-Barr virus in extranodal natural killer T-cell lymphoma. Leukemia. 2018. 10.1038/s41375-018-0324-5. 10.1038/s41375-018-0324-5 PMC6756073 30546078

[11] Gru AA, Haverkos BH, Freud AG, Hastings J, Nowacki NB, Barrionuevo C (2015). The Epstein-Barr virus (EBV) in T cell and NK cell lymphomas: time for a reassessment. Curr Hematol Malig Rep.

[12] Fox CP, Haigh TA, Taylor GS, Long HM, Lee SP, Shannon-Lowe C (2010). A novel latent membrane 2 transcript expressed in Epstein-Barr virus-positive NK- and T-cell lymphoproliferative disease encodes a target for cellular immunotherapy. Blood.

[13] Sadler RH, Raab-Traub N (1995). Structural analyses of the Epstein-Barr virus BamHI A transcripts. J Virol.

[14] Smith PR, de Jesus O, Turner D, Hollyoake M, Karstegl CE, Griffin BE (2000). Structure and coding content of CST (BART) family RNAs of Epstein-Barr virus. J Virol.

[15] de Jesus O, Smith PR, Spender LC, Elgueta Karstegl C, Niller HH, Huang D (2003). Updated Epstein-Barr virus (EBV) DNA sequence and analysis of a promoter for the BART (CST, BARF0) RNAs of EBV. J Gen Virol.

[16] Li Z, Xia Y, Feng L-N, Chen J-R, Li H-M, Cui J (2016). Genetic risk of extranodal natural killer T-cell lymphoma: a genome-wide association study. Lancet Oncol.

[17] Kojya S, Matsumura J, Ting L, Hongyo T, Inazawa J, Kirihata M (2001). Familial nasal NK/T-cell lymphoma and pesticide use. Am J Hematol.

[18] Chan JY, Ng AYJ, Cheng CL, Nairismagi ML, Venkatesh B, Cheah DMZ (2018). Whole exome sequencing identifies recessive germline mutations in FAM160A1 in familial NK/T cell lymphoma. Blood Cancer J.

[19] Song TL, Nairismagi ML, Laurensia Y, Lim JQ, Tan J, Li ZM (2018). Oncogenic activation of the STAT3 pathway drives PD-L1 expression in natural killer/T-cell lymphoma. Blood.

[20] Kucuk C, Jiang B, Hu X, Zhang W, Chan JK, Xiao W (2015). Activating mutations of STAT5B and STAT3 in lymphomas derived from gammadelta-T or NK cells. Nat Commun.

[21] Jiang L, Gu ZH, Yan ZX, Zhao X, Xie YY, Zhang ZG (2015). Exome sequencing identifies somatic mutations of DDX3X in natural killer/T-cell lymphoma. Nat Genet.

[22] Dobashi A, Tsuyama N, Asaka R, Togashi Y, Ueda K, Sakata S (2016). Frequent BCOR aberrations in extranodal NK/T-cell lymphoma, nasal type. Genes Chromosomes Cancer.

[23] Lee S, Park HY, Kang SY, Kim SJ, Hwang J, Lee S (2015). Genetic alterations of JAK/STAT cascade and histone modification in extranodal NK/T-cell lymphoma nasal type. Oncotarget..

[24] Koo GC, Tan SY, Tang T, Poon SL, Allen GE, Tan L (2012). Janus kinase 3-activating mutations identified in natural killer/T-cell lymphoma. Cancer Discov.

[25] Sim SH, Kim S, Kim TM, Jeon YK, Nam SJ, Ahn YO (2017). Novel JAK3-activating mutations in extranodal NK/T-Cell lymphoma, nasal type. Am J Pathol.

[26] Bouchekioua A, Scourzic L, de Wever O, Zhang Y, Cervera P, Aline-Fardin A (2014). JAK3 deregulation by activating mutations confers invasive growth advantage in extranodal nasal-type natural killer cell lymphoma. Leukemia.

[27] Kimura H, Karube K, Ito Y, Hirano K, Suzuki M, Iwata S (2014). Rare occurrence of JAK3 mutations in natural killer cell neoplasms in Japan. Leuk Lymphoma.

[28] Nairismagi M, Gerritsen ME, Li ZM, Wijaya GC, Chia BKH, Laurensia Y (2018). Oncogenic activation of JAK3-STAT signaling confers clinical sensitivity to PRN371, a novel selective and potent JAK3 inhibitor, in natural killer/T-cell lymphoma. Leukemia.

[29] Scuto A, Kujawski M, Kowolik C, Krymskaya L, Wang L, Weiss LM (2011). STAT3 inhibition is a therapeutic strategy for ABC-like diffuse large B-cell lymphoma. Cancer Res.

[30] Hong D, Kurzrock R, Kim Y, Woessner R, Younes A, Nemunaitis J (2015). AZD9150, a next-generation antisense oligonucleotide inhibitor of STAT3 with early evidence of clinical activity in lymphoma and lung cancer. Sci Transl Med.

[31] Huynh J, Chand A, Gough D, Ernst M (2019). Therapeutically exploiting STAT3 activity in cancer - using tissue repair as a road map. Nat Rev Cancer.

[32] Jo JC, Kim M, Choi Y, Kim HJ, Kim JE, Chae SW (2017). Expression of programmed cell death 1 and programmed cell death ligand 1 in extranodal NK/T-cell lymphoma, nasal type. Ann Hematol.

[33] Kim WY, Jung HY, Nam SJ, Kim TM, Heo DS, Kim CW (2016). Expression of programmed cell death ligand 1 (PD-L1) in advanced stage EBV-associated extranodal NK/T cell lymphoma is associated with better prognosis. Virchows Arch.

[34] Han L, Liu F, Li R, Li Z, Chen X, Zhou Z (2014). Role of programmed death ligands in effective T-cell interactions in extranodal natural killer/T-cell lymphoma. Oncol Lett.

[35] Kwong YL, Chan TSY, Tan D, Kim SJ, Poon LM, Mow B (2017). PD1 blockade with pembrolizumab is highly effective in relapsed or refractory NK/T-cell lymphoma failing l-asparaginase. Blood.

[36] Li X, Cheng Y, Zhang M, Yan J, Li L, Fu X (2018). Activity of pembrolizumab in relapsed/refractory NK/T-cell lymphoma. J Hematol Oncol.

[37] Lim JQ, Tang T, Cai Q-q, Tan D, Nairismagi M-L, Laurensia Y (2018). Recurrent PD-L1 structural rearrangements in natural killer/T cell lymphoma patients with complete response to PD-1 blockade therapy. bioRxiv.

[38] Bellone S, Buza N, Choi J, Zammataro L, Gay L, Elvin J (2018). Exceptional response to pembrolizumab in a metastatic, chemotherapy/radiation-resistant ovarian cancer patient harboring a PD-L1-genetic rearrangement. Clin Cancer Res.

[39] Van Allen EM, Golay HG, Liu Y, Koyama S, Wong K, Taylor-Weiner A (2015). Long-term benefit of PD-L1 blockade in lung cancer associated with JAK3 activation. Cancer Immunol Res.

[40] Ng SB, Selvarajan V, Huang G, Zhou J, Feldman AL, Law M (2011). Activated oncogenic pathways and therapeutic targets in extranodal nasal-type NK/T cell lymphoma revealed by gene expression profiling. J Pathol.

[41] Yan J, Ng SB, Tay JL, Lin B, Koh TL, Tan J (2013). EZH2 overexpression in natural killer/T-cell lymphoma confers growth advantage independently of histone methyltransferase activity. Blood.

[42] Sander S, Bullinger L, Klapproth K, Fiedler K, Kestler HA, Barth TF (2008). MYC stimulates EZH2 expression by repression of its negative regulator miR-26a. Blood.

[43] EZH2 mediates resistance to apoptosis in Nktl by activating Nfkb signaling through repression of TNFAIP3/A20 by H3K27 trimethylation. Blood 2013;122(21):1278.

[44] Yan J, Li B, Lin B, Lee PT, Chung TH, Tan J (2016). EZH2 phosphorylation by JAK3 mediates a switch to noncanonical function in natural killer/T-cell lymphoma. Blood.

[45] Cao Q, Gearhart MD, Gery S, Shojaee S, Yang H, Sun H (2016). BCOR regulates myeloid cell proliferation and differentiation. Leukemia.

[46] Zhang Y, Li C, Xue W, Zhang M, Li Z (2018). Frequent mutations in natural killer/T cell lymphoma. Cell Physiol Biochem.

[47] Lohr JG, Stojanov P, Lawrence MS, Auclair D, Chapuy B, Sougnez C (2012). Discovery and prioritization of somatic mutations in diffuse large B-cell lymphoma (DLBCL) by whole-exome sequencing. Proc Natl Acad Sci.

[48] Wang L, Wang H, Li PF, Lu Y, Xia ZJ, Huang HQ (2015). CD38 expression predicts poor prognosis and might be a potential therapy target in extranodal NK/T cell lymphoma, nasal type. Ann Hematol.

[49] Hari P, Raj RV, Olteanu H (2016). Targeting CD38 in refractory extranodal natural killer cell-T-cell lymphoma. N Engl J Med.

[50] Morschhauser F, Salles G, McKay P, Tilly H, Schmitt A, Gerecitano J (2017). Interim report from a phase 2 multicenter study of tazemetostat, an EZH2 inhibitor, in patients with relapsed or refractory b-cell non-hodgkin lymphomas. Hematol Oncol.

[51] Knutson SK, Wigle TJ, Warholic NM, Sneeringer CJ, Allain CJ, Klaus CR (2012). A selective inhibitor of EZH2 blocks H3K27 methylation and kills mutant lymphoma cells. Nat Chem Biol.

[52] Zeng D, Liu M, Pan J (2017). Blocking EZH2 methylation transferase activity by GSK126 decreases stem cell-like myeloma cells. Oncotarget.

[53] McCabe MT, Ott HM, Ganji G, Korenchuk S, Thompson C, Van Aller GS (2012). EZH2 inhibition as a therapeutic strategy for lymphoma with EZH2-activating mutations. Nature.

[54] Chan TSY, Li J, Loong F, Khong PL, Tse E, Kwong YL (2018). PD1 blockade with low-dose nivolumab in NK/T cell lymphoma failing L-asparaginase: efficacy and safety. Ann Hematol.

[55] Kim W-S, Eom HS, Yeh S-P, Cho S-G, Heo DS, Kim JS, Yao M, Zhu J, Gao G, Zhang L, Qi M, Huang H, Kwong YL (2018). Daratumumab monotherapy for patients with relapsed or refractory (R/R) natural killer/T-cell lymphoma (NKTCL), nasal type: an open-label, single-arm, multicenter phase 2 study. ASH Annual meeting Poster 1617.

